# Fabrication of functional ameloblasts from hiPSCs for dental application

**DOI:** 10.3389/fcell.2023.1164811

**Published:** 2023-06-29

**Authors:** Ka-Hwa Kim, Eun-Jung Kim, Hyun-Yi Kim, Shujin Li, Han-Sung Jung

**Affiliations:** ^1^ Division in Anatomy and Developmental Biology, Department of Oral Biology, Taste Research Center, Oral Science Research Center, BK21 FOUR Project, Yonsei University College of Dentistry, Seoul, Republic of Korea; ^2^ NGeneS Inc, Ansan-si, Republic of Korea

**Keywords:** human-induced pluripotent stem cell, dental epithelial cell, organoid, ameloblast, tooth formation capacity

## Abstract

Tooth formation relies on two types of dental cell populations, namely, the dental epithelium and dental mesenchyme, and the interactions between these cell populations are important during tooth development. Although human-induced pluripotent stem cells (hiPSCs) can differentiate into dental epithelial and mesenchymal cells, organoid research on tooth development has not been established yet. This study focused on the hiPSC-derived human ameloblast organoid (hAO) using a three-dimensional (3D) culture system. hAOs had similar properties to ameloblasts, forming enamel in response to calcium and mineralization by interaction with the dental mesenchyme. hAOs simultaneously had osteogenic and odontogenic differentiation potential. Furthermore, hAOs demonstrated tooth regenerative potential upon interaction with the mouse dental mesenchyme. Our findings provide new insights into a suitable hiPSC-derived dental source and demonstrate that hAOs can be beneficial not only for tooth regeneration but also for the study of various dental diseases for which treatment has not been developed yet.

## Introduction

Organoids are *in vitro* self-organizing, self-renewing, organ-like, and three-dimensional (3D) cellular structures that have the key characteristics of each organ. Organoids retain many structural and functional features, such as cell composition and tissue architecture, of their corresponding *in vivo* organs (Dutta et al., 2017). Human-induced pluripotent stem cells (hiPSCs) show great potential in advanced tissue engineering and cell therapies to replace damaged cells or regenerate organs, making them useful tools for disease modeling (Kolios and Moodley, 2013; Halevy and Urbach, 2014; Olgasi et al., 2020), since hiPSCs can differentiate into tissues in all three germ layers: ectoderm, mesoderm, and endoderm. hiPSCs are derived from somatic cells using gene transfer with reprogramming factors, and the characteristics of hiPSCs resemble those of embryonic stem cells (ESCs) morphologically, antigenically, and phenotypically. However, hiPSCs showed fewer ethical problems than ESCs and provided the advantages of patient-customized treatment (Soria-Valles and Lopez-Otin, 2016; Takahashi and Yamanaka, 2016).

Several hiPSC-derived organoids, such as the kidney (mesoderm) (Koning et al., 2020), liver (endoderm) (Guan et al., 2017; Prior et al., 2019; Olgasi et al., 2020), brain (ectoderm) (Lee et al., 2017), lung (ectoderm) (Dye et al., 2015), and intestinal (endoderm) (Rahmani et al., 2019) and retinal (ectoderm) (Bell et al., 2020) organoids, have been studied. However, there are still many limitations to the use of organoid culture systems, including variable sizes, low reproducibility, and limited maturation. Although research on organoids using existing stem cells is in progress, the differentiation of hiPSCs into dental epithelial cells and dental organoids is not elucidated yet.

Enamel-secreting ameloblasts are derived from dental epithelial stem cells (Binder et al., 2020). Although humans are unable to repair or regenerate enamel, due to the early loss of dental epithelial stem cells (Smith et al., 2017; Binder et al., 2020), epithelial stem cells of mouse incisors are maintained and generate ameloblasts throughout life (Kuang-Hsien Hu et al., 2014). It has been reported that Lgr5-positive cells, which play an important role in the maintenance and differentiation of stem cells in adult tissue, exist in the epithelium of the continuously growing mouse incisors (Suomalainen and Thesleff, 2010; Binder et al., 2020). Human dental stem cells, which can be obtained from postnatal teeth, extracted wisdom teeth, or exfoliated deciduous teeth (Morsczeck and Reichert, 2018), are promising for tooth repair because of their differentiation potential. Dental stem cells seem suitable for regenerating dental tissue, however, are still difficult to implement (Morsczeck and Reichert, 2018). Implant dentures that are used clinically to replace missing teeth do not always achieve the aesthetic and functional effects of natural teeth (Karimi Dastgerdi et al., 2020). Therefore, using stem cell technology to regenerate teeth with the same morphology and function as natural teeth is an ideal approach to solve the problem of tooth loss (Volponi et al., 2010).

Previous studies have shown that hiPSCs are differentiated into dental epithelial cell-derived ameloblasts (Kim et al., 2020; Kim et al., 2021) by the modulation of BMP signaling, which plays crucial roles in tooth development and regulates the maintenance and proliferation of the dental epithelium (Amand et al., 2000; Harvey et al., 2010; Hu et al., 2012; Zhang et al., 2017; Li et al., 2019). Unlike the two-dimensional (2D) culture system, hiPSC-derived ameloblast organoids (hAOs) based on 3D culture systems have not been reported. Attempts have been made to differentiate dental organoids using human dental follicle tissues, isolated from unerupted wisdom teeth (Hemeryck et al., 2022), in addition to deriving human dental pulp stem cells from mesenchymal cells (Jeong et al., 2020) and enamel spheroids from hiPSCs (Alghadeer et al., 2022). These studies developed a long-term expandable stemness organoid model from a human tooth, replicating molecular and functional features of the originating epithelial stem cell compartment. Although there have been several studies using stem cells that are related to human teeth, few studies on the differentiation of tooth organoids using hiPSCs have been conducted.

In this study, we established a suitable protocol for the differentiation of ameloblast organoids using a 3D culture system. Our results demonstrate that hiPSCs can differentiate into ameloblast organoids with ameloblast characteristics after 40 days. Ameloblast organoids from hiPSCs show ameloblast-like characteristics with calcium response and mineralization through interaction with the dental mesenchyme. This novel protocol could be used for tooth-related disease models and tooth regeneration studies.

## Materials and methods

All experiments were performed according to the guidelines of the Intramural Animal Use and Care Committee, Yonsei University College of Dentistry (2019-0306).

### Formation of hAOs

Between days 0 and 4, hiPSCs (10,000 cells/well) were cultured in StemFlex medium (Gibco BRL, NY, United States) using a U-bottom plate (S-BIO, NH, United States) to form embryoid bodies (EBs). Between days 4 and 8, EBs (192 EBs/100 mm^2^) were cultured in Dulbecco’s Modified Eagle Medium/Nutrient Mixture F-12 (DMEM/F-12, Gibco) containing ×1 N-2 supplement (Gibco), 0.1 mM RA (Sigma-Aldrich, MO, United States), and 50 ng/mL BMP4 (R&D Systems, Inc., MN, United States) in a fibronectin-coated dish (Advanced BioMatrix, CA, United States). Between days 8 and 12, the medium was replaced with (Keratinocyte Serum-Free Medium (K-SFM, Gibco), 100 ng/mL Noggin (PeproTech, NJ, United States), and 1 μg/mL EGF (PeproTech). Between days 12 and 26, the dissociated cells (40,000 cell/40 μL) were embedded in Matrigel (Corning, NY, United States) and cultured in Keratinocyte Basal Medium (KBM, Lonza, Switzerland) with WREFD media for differentiation, which was then modified to Clever’s organoid medium (Kretzschmar and Clevers, 2016) containing 50% Wnt3a conditional medium, 5% R-spondin1 conditional medium, 100 ng/mL EGF (PeproTech), 100 ng/mL FGF10 (PeproTech), and 10 µM dibenzazepine (Cayman, MI, United States) supplemented with 200 ng/mL Noggin (PeproTech), ×1 N-2 supplement (Gibco), ×1 B-27 supplement (Gibco), 10 mM nicotinamide (N0636, Sigma), 0.5 µM A83-01 (Tocris, United Kingdom), 1 mM N-acetylcysteine (Sigma), 10 µM Y-27632 (Cayman), and 1% P/S (Gibco). Finally, between days 26 and 40, the medium was replaced by removing Noggin and adding 100 ng/mL BMP4 (R&D Systems) for induction of differentiation and proliferation, and the media were changed every 3 days.

### Calcium imaging (Fluo-4 AM)

Calcium imaging was performed as previously described with modifications (Paez et al., 2007). In brief, hAOs were seeded in 35-mm confocal dishes (SPL Life Sciences, Korea) and waited 7–10 days until the epithelial cells were derived from hAOs. HaCaT cells (the immortalized human keratinocytes were used as a negative control) were seeded in 35-mm confocal dishes and waited for 2 days. Furthermore, C2C12 cells (the myoblast cell line was used as a positive control) were seeded in 35-mm confocal dishes, and the myogenic differentiation media (2% horse serum in DMEM) were changed for 4 days. To release the endogenous Ca^2+^ from the endoplasmic reticulum, the hAO-derived epithelial cells, HaCaT cells, and C2C12 cells were incubated in DPBS at 37°C for 30 min. The cells were loaded with 5 µM Fluo-4 AM (Invitrogen) in DPBS containing 0.01% Pluronic F-127 (Invitrogen) and incubated at 37 °C for 30 min. Before imaging, cells were washed four times with DPBS. The Fluo-4 fluorescence was excited at wavelengths of 494 nm every 1 s using a high-speed wavelength device. Images (512 × 512 pixels) were recorded using a Leica DMi8 confocal microscope. The basal Fluo-4 fluorescence level was recorded under a Ca^2+^-free condition for 10 s. CaCl_2_ solution (final concentration: 2 mM) was added to the confocal dish, and the change in fluorescence was immediately monitored continuously for 120 s. To minimize bleaching, the intensity of the excitation light and sampling frequency was kept as low as possible, and 30 cells were analyzed.

### *In vitro* osteogenic assay

The hAOs were induced in osteogenic differentiation medium (ODM, WREFD containing Noggin mixed with 100 nM dexamethasone, 10 nM β-glycerophosphate, 60 μM L-ascorbic acid, and 1.8 mM KH_2_PO_4_) in a 3D culture system for 14 days. The hAOs were seeded in a fibronectin-coated dish and cultured with ODM for 7 days. The epithelial cells were then spread from the hAOs (hAO-derived epithelial cells). For alkaline phosphotase (ALP; MK300, Takara) staining, the cells were fixed with 4% paraformaldehyde (PFA), washed with phosphate-buffered saline (PBS) and incubated in ALP working solution at 37°C for 45 min.

### Histology and immunostaining

For the paraffin section, the Matrigel was broken with a cold KBM (Lonza). The hAOs were fixed in 4% PFA for 30 min and dehydrated with graded ethanol solutions for 30 min. Then, the blocks were embedded into paraffin and made into 4-μm sections. For hematoxylin and eosin (H&E) staining, the sections were rehydrated and then were stained. For immunofluorescence, antigen retrieval was achieved by citrate buffer, pH 6.0 at 121°C or 10 μg/mL proteinase K at 37°C. After cooling down, slides were treated with blocking solution, and the following first antibodies were used: OCT3/4 (Santa Cruz, TX, United States), OTX2 (R&D systems), Brachyury (R&D systems), SOX17 (R&D systems), PCNA (Abcam, CAM, United Kingdom), cleaved caspase-3 (Cell Signaling Technology, MA, United States), E-cad (BD biosciences, CA, United States), cytokeratin 14 (Abcam), p63 (GeneTex, CA, United States), collagen IV (Abcam), PITX2 (Abnova, Taiwan), DLX3 (Invitrogen, OR, United States), amelogenin (AMELX; Santa Cruz), and ameloblastin (AMBN; Biorbyt, UK, dentin sialoprotein (DSP; Santa Cruz), periostin (Abcam), cementum protein (CEMP1; Abcam), and human leukocyte antigen (hLA; Abcam). For visualization, anti-mouse or rabbit IgG conjugated with Alexa Fluor 488 or 555 dye (Invitrogen) was applied and observed under a confocal laser microscope (DMi8; Leica, Germany).

### Real-time quantitative polymerase chain reaction

The total RNA was extracted using TRIzol^®^ reagent (Thermo Fisher Scientific, MA, United States). The extracts were reverse-transcribed using Maxime RT PreMix (iNtRON, Korea). RT-qPCR was performed using a StepOnePlus Real-Time PCR System (Applied Biosystems, United States). The amplification program consisted of 40 cycles of denaturation at 95°C for 15 s and annealing at 63°C for 30 s. The expression levels of each gene are expressed as normalized ratios against the B2M housekeeping gene. The primers used for RT-qPCR are listed in Table 1.

**TABLE 1:** primers used for RT-qPCR.

Gene	Sequence (5′–3′)
*CK14*	F-CAT GAG TGT GGA AGC CGA CAT
	R-GCC TCT CAG GGC ATT CAT CTC
*PITX2*	F-CGC TCC CTC TTT CTC CAT TT
	R-AGG CCA CTT TCC AGA GGA AC
*SHH*	F-CCA ATT ACA ACC CCG ACA TC
	R-AGT TTC ACT CCT GGC CAC TG
*DLX3*	F-CCC TGC CCG AGT CTT CTG TC
	R-CCC CGT ATT GCC GGT AGG AG
*AMELX*	F-AGC ATA AGG CCA CCG TAC CC
	R-GCC AGG AAC GGG CAT CAT TG
*COL1A1*	F-CCC ACC AAT CAC CTG CGT AC
	R-GGT TTC CAC ACG TCT CGG TC
*RUNX2*	F-CCC AGT ATG AGA GTA GGT GTC C
	R-GGG TAA GAC TGG TCA TAG GAC C
*Osterix*	F-CTG CCC ACC TAC CCA TCT GA
	R-ACC ACT CCC TTC TAG CTG CC
*DSPP*	F-GCC ATT CCA GTT CCT CAA AGC
	R-CAT GCA CCA GGA CAC CAC TT
*DMP1*	F-ATG CCT ATC ACA ACA AAC C
	R-CTC CTT TAT GTG ACA ACT GC
*GAPDH*	F-GAC GCT GGG GCT GGC ATT G
	R-GCT GGT GGT CCA GGG GTC

F, forward primer; R, reverse primer.

### Recombination hAO with the dental mesenchyme

A hAO was recombined with the dental mesenchyme isolated from the ICR mouse mandibular tooth germs at embryonic day 14 in DMEM (Gibco) supplemented with 20% FBS and 1% P/S for 2 days.

### Kidney transplantation

Recombinants (n = 15) were implanted under the renal capsule of an immunocompromised PN 6-week male mouse (BALB/C nu/nu purchased from Nara Biotech Co.) for 12 weeks. Since immunocompromised mice do not have a thymus, they are commonly used in xenotransplantation experiments and tissue calcification. A total of five mice were randomly selected for the recombinant transplantation experiment for calcification. Mice that died due to transplantation were excluded; however, there was no exclusion in this experiment. The mice were incised on the dorsal side, and the kidney was collected. Using the tungsten needle, a small hole at the renal capsule covering the kidney was made to insert recombinants. Once this procedure was completed, the kidney was transferred back inside the abdominal cavity, and the incision was sutured. Furthermore, 16 weeks after the surgery, the mice were euthanized in a CO_2_ chamber to collect the kidney. The collected kidney was fixed in 4% paraformaldehyde and scanned using a micro-CT scanner. Samples were decalcified with 10% EDTA at 50°C, and histological analysis was performed after reviewing.

### Micro-CT

Three-dimensional reconstructed computed tomography images were obtained by scanning calcified teeth using micro-computed tomography (n = 10) (Micro-CT, Skyscan, Belgium).

### Statistical analysis

All results are presented as the means and standard deviations of at least three independent experiments for *in vitro* assays (n = 3). Comparisons between two groups were analyzed using Student’s *t*-test. Experiments were analyzed by one-way or two-way analysis of variance (ANOVA) followed by Tukey’s *post hoc* test. A *p*-value < 0.05 was considered statistically significant. Non-significant values have been shown as ns in the Results section, while *, **, and *** describe *p*-values <0.05, 0.01, and 0.001, respectively. All statistical analyses were performed using GraphPad Prism 9 software.

## Results

### Generation of hAOs

**FIGURE 1:**
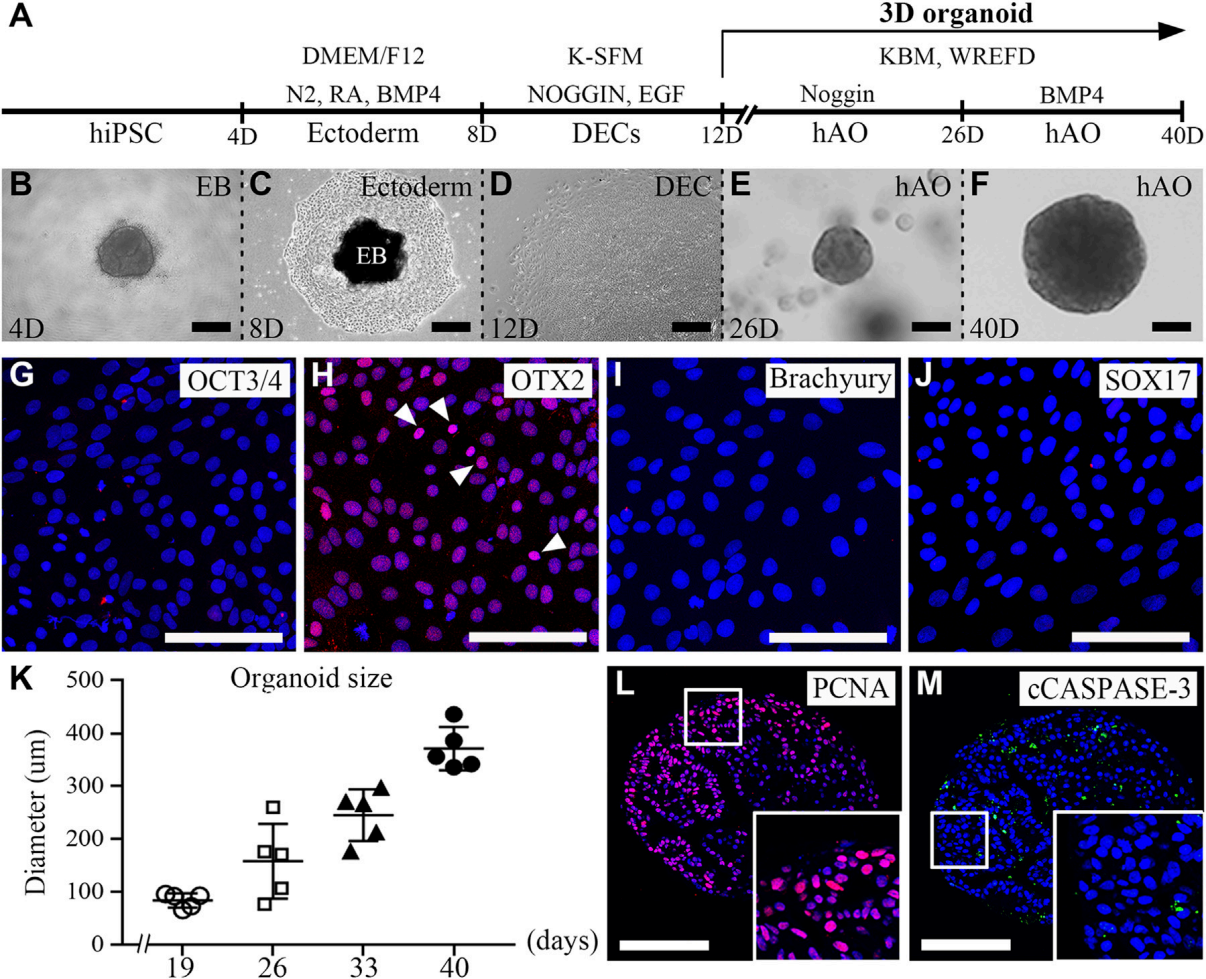
Generation of hiPSC-derived ameloblast organoids. **(A)** Schematic overview of the procedures for generating hiPSC-derived organoids. **(B)** Between days 0 and 4, hiPSCs were cultured in StemFlex medium using a U-bottom plate to form EBs. **(C)** Between days 4 and 8, EBs were cultured in a fibronectin-coated dish with DMEM/F12 medium supplemented with N2, RA, and BMP4. **(D)** Between days 8 and 12, the medium was replaced with K-SFM, Noggin, and EGF. **(E)** Between days 12 and 26, the dissociated cells were embedded into Matrigel and cultured in KBM including Wnt3a, R-spondin1, EGF, FGF10, dibenzazepine, Noggin, nicotinamide, A83-01, N-acetylcysteine, and Y-27632 (WREFD with Noggin). **(F)** Between days 26 and 40, the medium was replaced by removing Noggin and adding BMP4 (WREFD with BMP4). **(B–F)** Representative bright field images of cells at each time period. **(G–J)** Immunocytochemistry of OCT3/4, OTX2, Brachyury, and SOX17 in ectodermal cells cultured for 8 days. Only OTX2 was expressed in ectodermal cells. **(K)** The diameter of organoids was plotted (n = 5), and hiPSC-derived organoids gradually increased in size to approximately 400 μm. **(L, M)** Immunohistochemistry of PCNA and cCaspase-3 in organoids. PCNA was expressed in most organoid cells. In contrast, cCaspase-3 was rarely expressed in organoids. Scale bars: E, F, G, H, I, L, and M = 100 μm, B and D = 200 μm. TOPRO-3 (blue) was used to label the nuclei. White arrow heads: expression cells.

To establish hAOs, hiPSCs were differentiated into ectodermal cells and dental epithelial cells and embedded into Matrigel (Figure 1A). We modified the previous protocol to differentiate hiPSCs into human dental epithelial cells (Kim et al., 2021), and the previous organoid medium (Kretzschmar and Clevers, 2016) was also modified to differentiate hiPSC-derived dental epithelial cells into the dental epithelial organoid. hiPSCs were cultured in a U-bottom plate for 4 days to form EBs (Figure 1B). The EBs were differentiated into the ectoderm using BMP4 for 4 days in the fibronectin-coated dish. The differentiated cells from EBs were of a cuboidal shape, which is a typical epithelial cell shape (Figures 1C, D). To identify the characteristics of differentiated cells after 8 days (Figure 1C), immunofluorescence analysis was performed with OCT3/4 (a stem cell marker, Figure 1G), OTX2 (an ectoderm marker, Figure 1H), Brachyury (a mesoderm marker, Figure 1I), and SOX17 (an endoderm maker, Figure 1J). However, the differentiated cells expressed only OTX2 and did not express OCT3/4, brachyury, or SOX17. These results demonstrated that the differentiated cells from hiPSCs after 8 days show the characteristics of the definitive ectoderm. For differentiation from ectodermal cells to dental epithelial cells (2D), ectodermal cells were cultured in K-SFM dental epithelial cell differentiation medium containing Noggin and EGF supplements for 8–12 days, which followed the dental epithelial cell differentiation protocol of our previous study.

After 12 days, for organoid differentiation, the differentiated dental epithelial cells were embedded in Matrigel and cultured for 28 days in KBM including WREFD [Wnt3a (stem cell maintenance), R-spondin1 (Wnt agonist), EGF (maintenance of stem cell niche), FGF10 (dental epithelial stem cell formation and maintenance), dibenzazepine (Notch inhibitor)], supplemented with nicotinamide (ROCK inhibitor), A83-01 (TGF-β inhibitor), N-acetylcysteine (stem cell maintenance), and Y-27632 (ROCK inhibitor) (Harada et al., 2002; Bartfeld et al., 2015; Kretzschmar and Clevers, 2016; Meng et al., 2018). The hiPSC-derived organoids were cultured in WREFD with Noggin for stem cell maintenance from 12 to 26 days (Figure 1E). For 14 days thereafter, the medium was replaced by removing Noggin and adding BMP4 to induce organoid differentiation and dental epithelial development (Kim et al., 2021). After 40 days, the differentiated hiPSC-derived organoids demonstrated spherical morphologies (Figure 1F). The hiPSC-derived organoids cultured for 40 days were stable and gradually increased in size to approximately 400 μm (Figure 1K). To confirm the proliferation and apoptosis of hiPSC-derived organoids, immunofluorescence analysis was performed with PCNA (a proliferation marker, Figure 1L) and cleaved caspase-3 (cCaspase-3, an apoptotic marker, Figure 1M) (Figures 1L, M). Most of the cells of the hiPSC-derived organoids were PCNA-positive. In contrast, cCaspase-3-positive cells were not present. These results demonstrated that the protocol for differentiating hAOs from hiPSC with high proliferation was successfully established with Noggin–BMP modulation for 40 days.

### Characterization of hAOs

**FIGURE 2:**
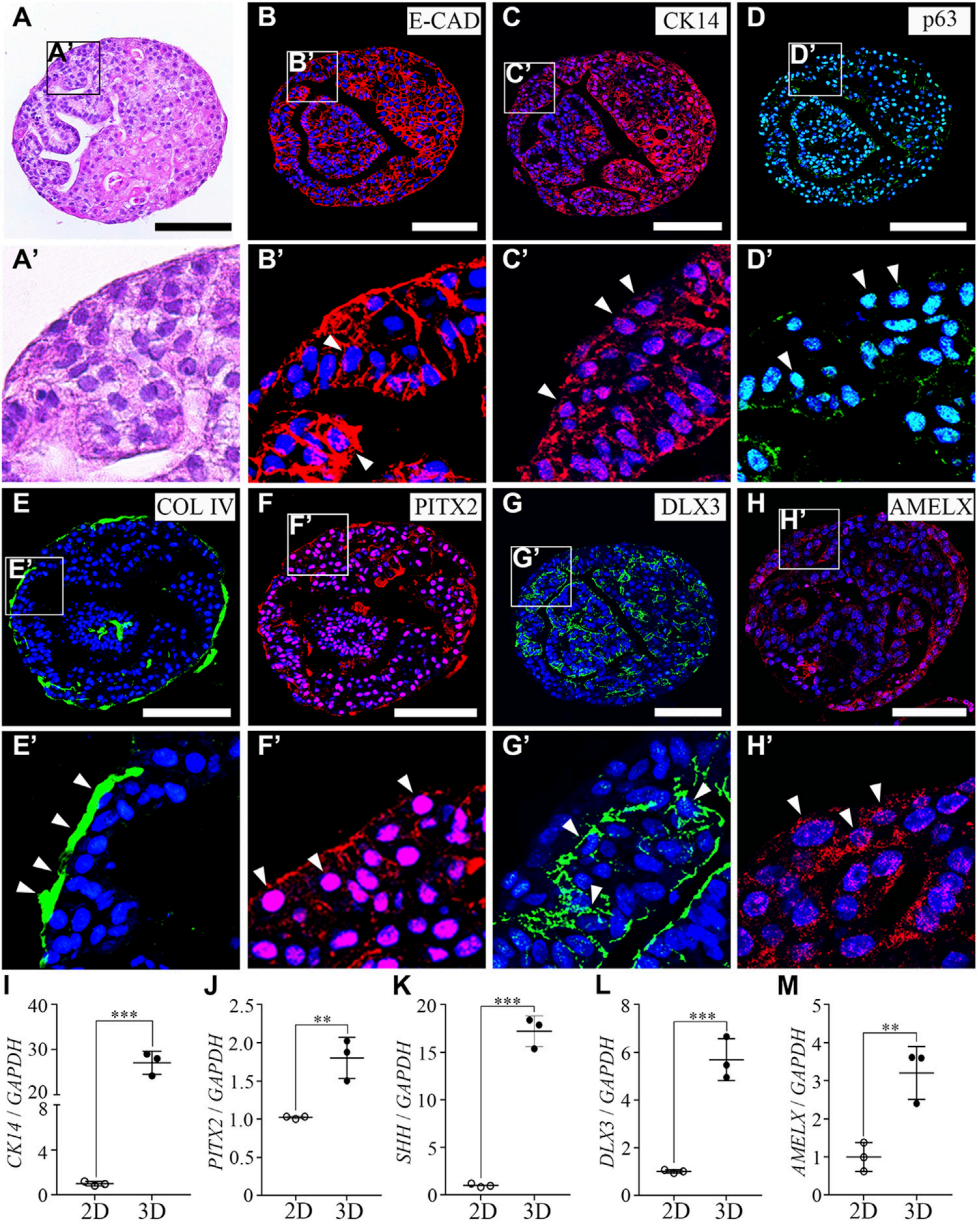
Characterization of hiPSC-derived ameloblast organoids. **(A)** H&E staining of paraffin sections of hiPSC-derived organoids. The round organoids displayed dense and complex morphologies. Organoids consisted of one cell layer in the outermost layer, a dense inner structure, and budding structures that are irregularly entangled inward. **(B, C)** Immunostaining of epithelial cell markers, E-CAD and CK14, **(D)** a basal epithelial cell marker, p63, **(E)** a basement membrane marker, Col IV, **(F)** a dental epithelial cell marker, PITX2, and **(G, H)** ameloblast markers, DLX3 and AMELX in organoids. (B′–H′) Higher magnification of the regions of interest shown in **(B–H)**. **(I**–**M)** mRNA expression of *CK14*, *PITX2*, *SHH*, *DLX3*, and *AMELX* was analyzed by RT-qPCR. On comparing the 2D (hiPSC-derived dental epithelial cells) and 3D cultures (hiPSC-derived organoids), the RNA expression levels of epithelial cell markers, dental epithelial cell markers, and ameloblast markers were significantly increased in the 3D culture. All experiments were performed in triplicate. Data are presented as means ± standard deviations (SD). TOPRO-3 (blue) was used to label the nuclei. Scale bar = 100 μm. White arrow heads: expression cells.

To characterize the hiPSC-derived organoids, we performed histology analysis using H&E staining. The round hiPSC-derived organoids displayed a dense and complex morphology. In addition, the hiPSC-derived organoids consisted of one cell layer in the outermost layer, with dense structures found inside the hiPSC-derived organoids, and the budding structures were irregularly entangled inward (Figure 2A). Furthermore, an immunofluorescence analysis was performed with E-CAD (Figure 2B) and CK14 (Figure 2C) as epithelial cell markers, p63 (Figure 2D) as a basal epithelial cell marker, collagen IV (COL IV, Figure 2E) as a basement membrane marker, PITX2 (Figure 2F) as a dental epithelial cell marker, and DLX3 (Figure 2G) and AMELX (Figure 2H) as ameloblast markers in hiPSC-derived organoids. Epithelial cell markers, such as E-CAD, CK14, p63, and PITX2 were expressed in most cells of the hiPSC-derived organoids (Figures 2B–D, F). COL IV, a basement membrane marker, was expressed at the border between the organoid and the Matrigel (Figure 2E). Furthermore, while DLX3 was mainly expressed in the inward budding of hiPSC-derived organoids (Figure 2G), AMELX expression was observed more toward the outer layer of the organoids (Figure 2H).

To compare mRNA expression levels of hiPSC-derived dental epithelial cells (2D culture) and hiPSC-derived organoids (3D culture), RT-qPCR analysis was performed with *CK14*, *PITX2*, and *SHH* (dental epithelial cell markers, Figures 2I–K) and *DLX3* and *AMELX* (ameloblast markers, Fig. L, M). Compared to the 2D cultures, the RNA expression levels of dental epithelial markers and ameloblast markers were more significantly increased in the 3D culture. Collectively, our data indicated that organoids derived from hiPSCs have characteristics of not only dental epithelial cells but also ameloblasts using the 3D culture. Henceforth, it is appropriate to refer to organoids differentiated from hiPSCs with ameloblast characteristics as hiPSC-derived hAOs.

### The effect of hAOs on calcium response and osteogenic differentiation

**FIGURE 3:**
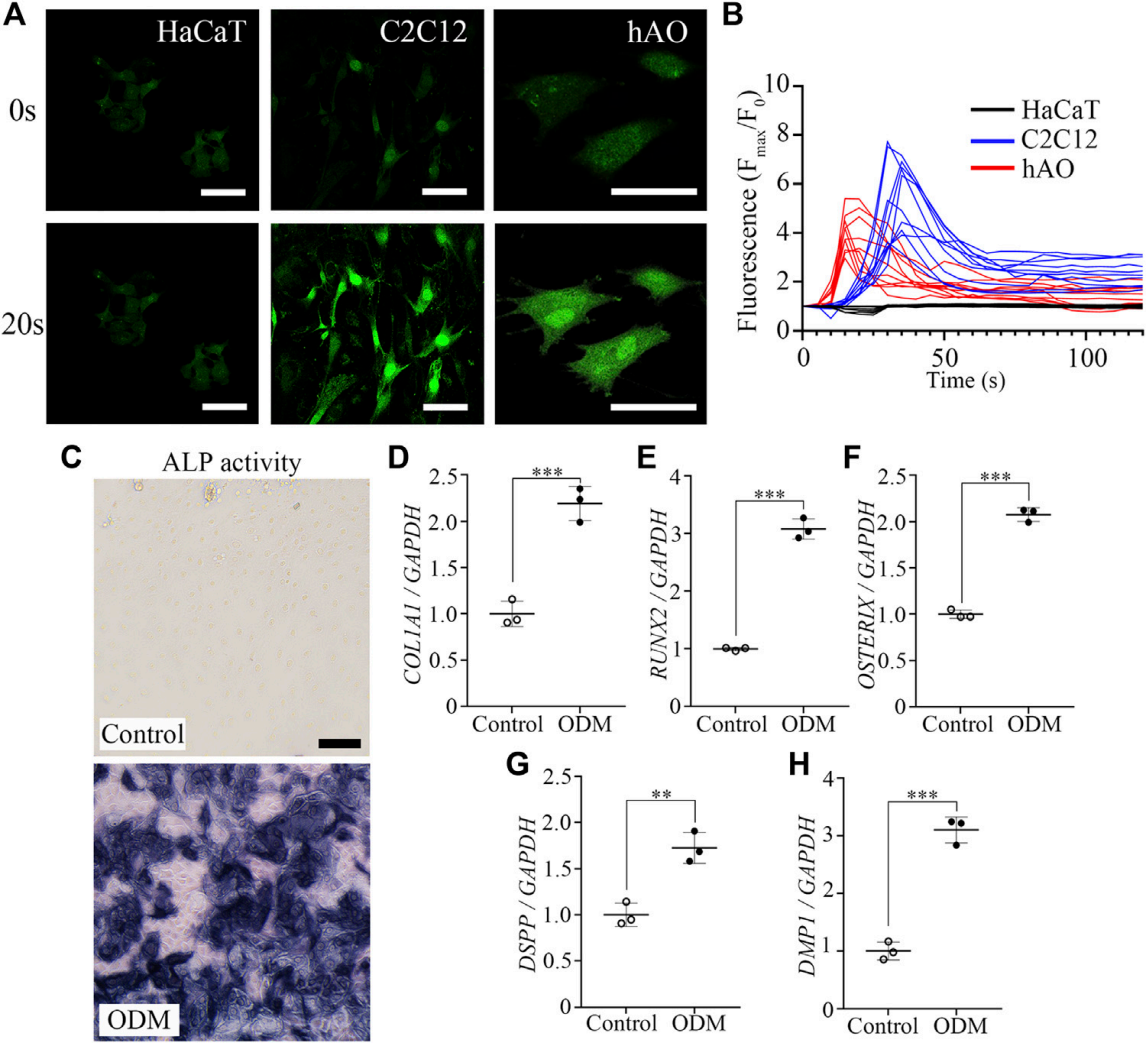
Functional assessment of the osteogenic and odontogenic potential of hAOs. **(A)** Representative image of the calcium response of hAO-derived dental epithelial cells, HaCaT cells, and C2C12 cells at 0 s and 20 s recording time. Intracellular Ca^2+^ was indicated by Fluo-4 AM (green). After being stimulated by CaCl_2_ (2 mM), fluorescence intensity remarkably increased. Scale bar = 50 μm. **(B)** The peak of the F/F0 (F: fluorescence intensity; F0: mean fluorescence intensity before stimulation) ratio was approximately recorded from 18 s to 30 s and gradually decreased during 120 s observation (n = 10). **(C)** ALP activity of hAO-derived epithelial cells cultured in control (WREFD with BMP4) or ODM for 21 days. Compared to the control group, ALP activity was increased in ODM. **(D–F)** The relative mRNA expression levels of osteogenic markers, *COL1A1*, *RUNX2*, and *Osterix*, were significantly increased in the ODM group compared to the control. **(G, H)** The relative mRNA expression levels of odontogenic markers, including DSPP and DMP1, were significantly increased in the ODM group compared to the control group. All experiments were performed in triplicate. Data are presented as the means ± standard deviations (SD). Scale bar: A = 50 μm; C = 100 μm.

Since the mineralization of the enamel matrix requires large amounts of intracellular calcium and extracellular phosphate, we investigated the capability of hAO-derived dental epithelial cells for calcium influx and the ALP activity level (one of the first functioning genes during calcification). Calcium imaging was performed to evaluate the Ca^2+^ influx capacity of hAO-derived dental epithelial cells and compared with HaCaT (an immortalized non-keratinized cell line) and C2C12 cells (a myoblast cell line). Fluo-4 AM (intracellular calcium indicator) was applied to highlight the Ca^2+^ influx. The oscillation of fluorescence intensity was monitored for 120 s after stimulating with CaCl_2_ solution (2 mM into final concentration) for 10 s of recording time. The fluorescence intensity of hAO-derived epithelial cells was significantly increased to 20 s of recording time compared to 0 s, which is similar to that of C2C12 cells. However, the fluorescence intensity of HaCaT cells was not changed during the recording time (Figure 3A). The peaks of the F/F0 ratio (F: fluorescence intensity; F0: mean fluorescence intensity before stimulation) of C2C12 and hAO-derived epithelial cells were observed from 18 s to 30 s of recording time and progressively decreased to the basal level. However, the peak of the F/F0 ratio of HaCaT cells was not shown (Figure 3B). These results revealed that hAO-derived dental epithelial cells have the Ca^2+^ influx capacity for mineralization.

The ALP activity of hAO-derived dental epithelial cells was also analyzed after 21 days of osteogenic induction. The intensive ALP staining was observed in the osteogenic differentiation medium (ODM) group compared to the control (WREFD with BMP4), indicating that the ALP activity of hAO-derived dental epithelial cells remarkably increased upon osteogenic induction (Figure 3C). After osteogenic differentiation, the expression of essential osteogenic differentiation genes in hAO-derived cells was analyzed using RT-qPCR. The relative mRNA expression level of *COL1A1*, *RUNX2*, and *Osterix* was significantly increased in the ODM group compared to the control (Figures 3D–F). Furthermore, the osteogenic induction also increased the mRNA expression level of odontogenic differentiation genes, including *DSPP* and *DMP1*, by stimulation from ODM (Figures 3G, H). These results demonstrated that hAOs simultaneously harbored potential for osteogenic and odontogenic differentiation and have the capacity for the epithelial–mesenchymal interaction.

### Odontogenic induction potential of hAOs

**FIGURE 4:**
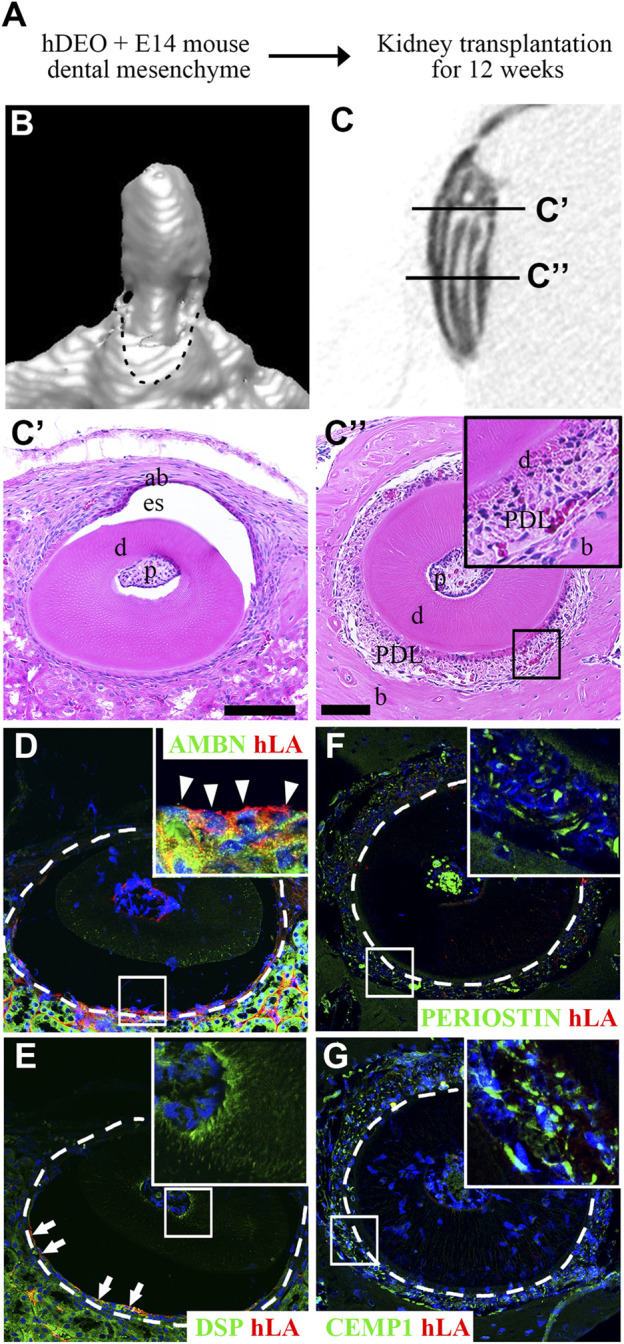
Odontogenic induction potential of hAOs. **(A)** Diagram of the recombinant of hAOs and dental mesenchyme from the mouse mandibular tooth germs at E14 followed by *in vivo* culture for 2 days and transplantation into the kidney capsule after 12 weeks to determine the odontogenic capacity of hAOs. **(B, C)** Micro-CT analysis of the mineralized tooth from the transplants. **(B)** 3D reconstruction images. **(C)** View of the sagittal section of the calcified tooth. (C′) The crown of the calcified tooth consists of ameloblast (ab), enamel space (es), dentin **(D)**, and pulp (p). (C″) The root of the calcified tooth consists of pulp (p), dentin **(D)**, periodontal ligament (PDL), and bone **(B)**. **(D, E)** In the crown, immunofluorescence analysis was performed for an ameloblast marker, AMBN, an odontoblast marker, DSP, and hLA. **(D)** Only AMBN-positive cells are co-localized with hLA. **(E)** DSP-positive cells were not co-localized with hLA. **(F, G)** In the root, immunofluorescence analysis was performed for a periodontal ligament marker, periostin, a cementum marker, CEMP1, and hLA. The expression of both periostin and CEMP1 was not co-localized with hLA. TOPRO-3 (blue) was used to label the nuclei. Scale bar = 100 μm. White arrow heads: co-localized cells. White arrows: hLA-positive cells.

To determine the odontogenic capacity of hAOs, the recombination of hAOs with the dental mesenchyme from the mouse mandibular tooth germs at E14 was cultured *in vitro* for 2 days and was transplanted into the kidney capsule of nude mice for 12 weeks (Figure 4A). The mineralization of the recombinant was observed as indicated by the micro-CT images of the 3D reconstruction (Figure 4B) and the sagittal section (Figure 4C). H&E staining revealed the structure of the mineralized tissue, including the dentin, enamel space, pulp, ameloblasts, and bone of the crown (Figure 4C’) and dentin, periodontal ligament (PDL), and bone of the root (Figure 4C”). Furthermore, the calcified tooth showed the co-localized expression of AMBN (an ameloblast marker, ameloblastin) and hLA (human leukocyte antigen) (Figure 4D). However, Dsp (an odontoblast marker, dentin sialoprotein, Figure 4E), periostin (a PDL marker, Figure 4F), and CEMP1 (a cementoblast marker, Figure 4G) did not co-localize with hLA. Therefore, hAOs differentiated into ameloblasts, secreted enamel, and had the capacity to form mineralized teeth by receiving signals from the mouse dental mesenchyme.

## Discussion

Because ameloblasts exist only during tooth development to deposit tooth enamel, enamel regeneration is impossible (Smith et al., 2017; Binder et al., 2020). In several previous tooth organoid studies, dental organoids were derived from stem cells present in human teeth. However, very few studies have differentiated hiPSCs into dental epithelial cells with ameloblast characteristics.

Previous studies demonstrated that dental epithelial cells differentiated from mouse iPSCs (Arakaki et al., 2012; Kim et al., 2019; Kim et al., 2020) as well as hiPSCs (Cai et al., 2013; Kim et al., 2021). These dental epithelial cells differentiated from hiPSCs, when co-cultured with human dental pulp stem cells (Kim et al., 2020), or recombined with the mouse dental mesenchyme (Kim et al., 2019; Kim et al., 2020; Kim et al., 2021), possessed not only osteogenic but also tooth formation capabilities and formed tooth-like structures. Furthermore, in recent studies, dental organoids were differentiated from the dental follicle isolated from unerupted wisdom teeth (Hemeryck et al., 2022), human dental pulp stem cell derived from the mesenchymal cell (Jeong et al., 2020), and enamel spheroids derived from hiPSCs (Alghadeer et al., 2022). However, the protocol for the differentiation of the dental epithelial organoid from hiPSCs has not been established yet. In this study, we established a novel protocol for deriving dental epithelial organoids from hiPSCs with ameloblast characteristics (hAO) using a 3D culture system for 40 days. First, 2D-cultured dental epithelial cells were differentiated for 12 days using the EB formation method from hiPSCs (Kim et al., 2021), and then the dental epithelial cells obtained from hiPSCs were further cultured in Matrigel for 28 days to differentiate them into a 3D culture of hAOs.

The hiPSC-derived dental epithelial cells were cultured in Matrigel with an optimized medium, and to further differentiate the hAOs into the ameloblast lineage, exogenous BMP4 was added to enhance cell proliferation and promote ameloblast characteristics. BMP signaling plays important multiple roles during tooth development, such as ectodermal differentiation and mediation of epithelial–mesenchymal interactions (Amand et al., 2000; Harvey et al., 2010; Hu et al., 2012; Zhang et al., 2017; Li et al., 2019). Therefore, hAOs have the characteristics of not only dental epithelial cells but also ameloblasts with DLX3 and AMELX expression.

Enamel is the hardest and most mineralized tissue in the body composed primarily of calcium. Ameloblasts are highly specialized epithelial cells that play an important role in mineralization of tooth enamel (Okumura et al., 2010; Nurbaeva et al., 2017). Matured ameloblasts mediate the secretion of Ca^2+^ from the blood to sites of mineralization. In the present study, hAOs from hiPSCs responded to calcium influx and showed physiologically functional ameloblast characteristics.

To investigate the ability of hAOs to induce osteogenic lineages, hAO-derived cells were cultured in ODM for 21 days. The differentiated cells with ODM stimulation showed a high ALP activity, and RNA levels of not only *COL1a1*, *RUNX2*, and *Osterix* but also *DSPP* and *DMP1* were increased. During tooth development, amelogenesis begins after dentinogenesis based on the epithelial–mesenchymal interaction (He et al., 2010; Nanci, 2018). *Dspp* is transiently expressed in early ameloblasts and is associated with ameloblast differentiation, physiology, and enamel biomineralization (Park et al., 2018). Furthermore, *DMP1* expression was observed in the ameloblast layer during the crown stage (Martinez et al., 2009). Therefore, hAOs have the capability of not only epithelial–mesenchymal interactions but also osteogenic induction.

Additionally, because the outer layer of hAOs is composed of Col IV, hAOs can recombine with and interact with the E14.5 mouse tooth mesenchyme. Co-localization of AMBN and hLA was observed in calcified teeth after 12 weeks, demonstrating that hAOs of human origin contributed to amelogenesis. Thus, these findings demonstrated that hAOs have biological functions that contribute not only to osteogenesis but also to odontogenic differentiation.

**FIGURE 5:**
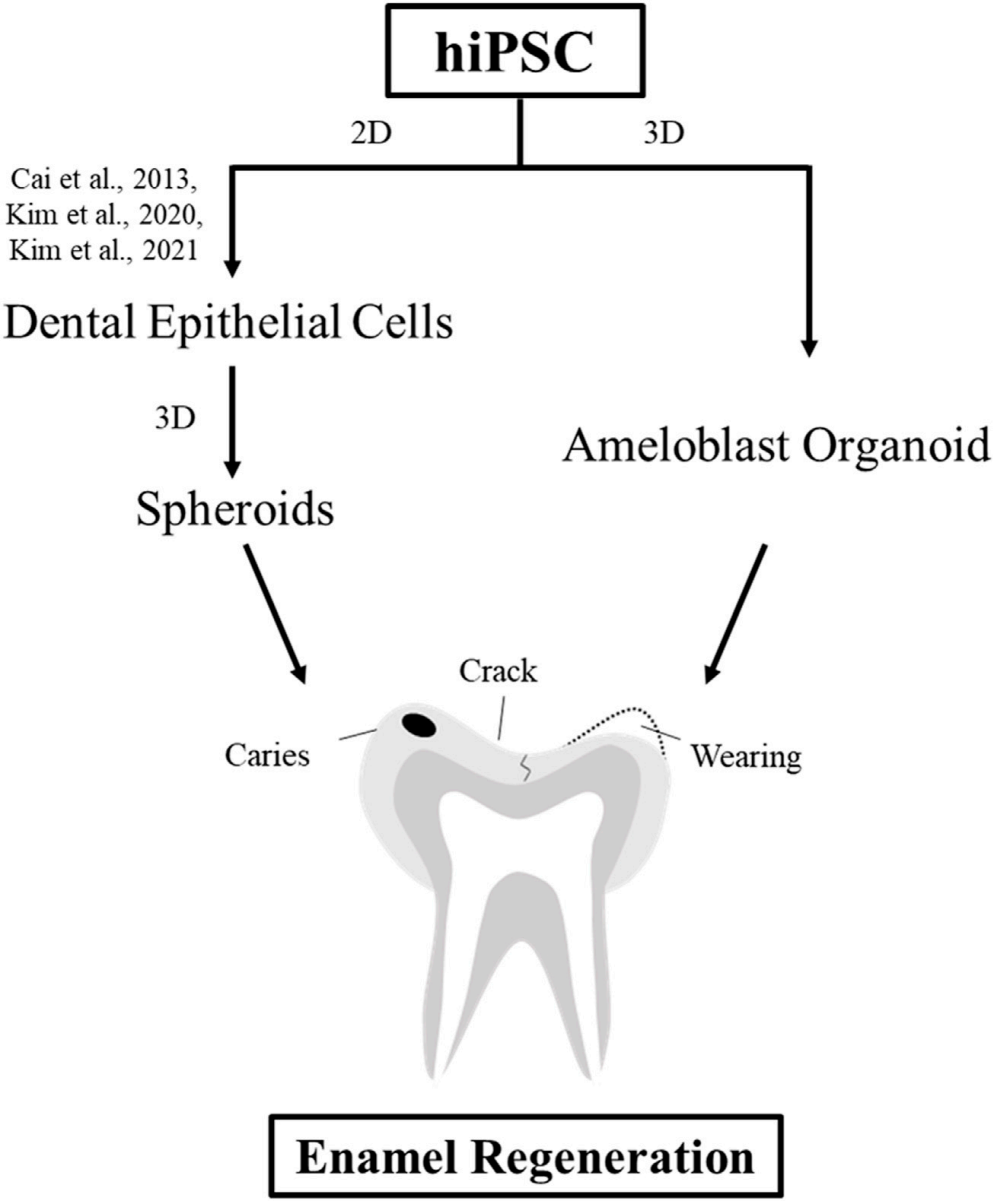
Schematic diagram of the strategies for enamel regeneration. Suitable sources of dental epithelial cells for enamel regeneration to repair enamel damage are expanded in the culture to generate a sufficient number of cells using either 2D or 3D culture systems. Dental epithelial cells were differentiated upon modulation of the signaling pathway in the 2D monolayer culture system. Dental epithelial cells derived from hiPSCs were cultured in a suspension to form spheroids. In this study, ameloblast organoid derived from hiPSCs (hAO) were differentiated upon Noggin–BMP modulation using the 3D culture system after 40 days and recombined with the mouse dental mesenchyme.

In conclusion, we established the first protocol for propagating hiPSC-derived hAOs (Figure 5). Our new tooth organoid model has the potential to differentiate into ameloblasts with high odontogenic ability to generate enamel. In order to use it for clinical tooth regenerative therapy in the future, it is necessary to study the mechanism and function for restoration of bioengineered teeth. This study contributes to the promising future of regenerative medicine by replacing 2D culture systems with more advanced 3D systems as a source of bioengineered dental epithelial cells using patient-specific cells.

## Data Availability

The original contributions presented in the study are included in the article/Supplementary Material; further inquiries can be directed to the corresponding author.
